# Mapping of Schistosomiasis and Soil-Transmitted Helminths in Namibia: The First Large-Scale Protocol to Formally Include Rapid Diagnostic Tests

**DOI:** 10.1371/journal.pntd.0003831

**Published:** 2015-07-21

**Authors:** José Carlos Sousa-Figueiredo, Michelle C. Stanton, Stark Katokele, Moses Arinaitwe, Moses Adriko, Lexi Balfour, Mark Reiff, Warren Lancaster, Bruce H. Noden, Ronnie Bock, J. Russell Stothard

**Affiliations:** 1 Centro de Investigação em Saúde de Angola (Health Research Center in Angola), Rua direita do Caxito, Hospital Provincial, Bengo, Angola; 2 Department of Life Sciences, Natural History Museum, Wolfson Wellcome Biomedical Laboratories, London, United Kingdom; 3 Department of Parasitology, Liverpool School of Tropical Medicine, Liverpool, United Kingdom; 4 Namibia Ministry of Health and Social Services, Windhoek, Namibia; 5 Vector Control Division, Ministry of Health, Kampala, Uganda; 6 The END Fund, New York, New York, United States of America; 7 Department of Entomology & Plant Pathology, Oklahoma State University, Stillwater, Oklahoma, United States of America; 8 Department of Biological Sciences, University of Namibia, Windhoek, Namibia; Swiss Tropical and Public Health Institute, SWITZERLAND

## Abstract

**Background:**

Namibia is now ready to begin mass drug administration of praziquantel and albendazole against schistosomiasis and soil-transmitted helminths, respectively. Although historical data identifies areas of transmission of these neglected tropical diseases (NTDs), there is a need to update epidemiological data. For this reason, Namibia adopted a new protocol for mapping of schistosomiasis and geohelminths, formally integrating rapid diagnostic tests (RDTs) for infections and morbidity. In this article, we explain the protocol in detail, and introduce the concept of ‘mapping resolution’, as well as present results and treatment recommendations for northern Namibia.

**Methods/Findings/Interpretation:**

This new protocol allowed a large sample to be surveyed (N = 17 896 children from 299 schools) at relatively low cost (7 USD per person mapped) and very quickly (28 working days). All children were analysed by RDTs, but only a sub-sample was also diagnosed by light microscopy. Overall prevalence of schistosomiasis in the surveyed areas was 9.0%, highly associated with poorer access to potable water (OR = 1.5, *P*<0.001) and defective (OR = 1.2, *P*<0.001) or absent sanitation infrastructure (OR = 2.0, *P*<0.001). Overall prevalence of geohelminths, more particularly hookworm infection, was 12.2%, highly associated with presence of faecal occult blood (OR = 1.9, *P*<0.001). Prevalence maps were produced and hot spots identified to better guide the national programme in drug administration, as well as targeted improvements in water, sanitation and hygiene. The RDTs employed (circulating cathodic antigen and microhaematuria for *Schistosoma mansoni* and *S*. *haematobium*, respectively) performed well, with sensitivities above 80% and specificities above 95%.

**Conclusion/Significance:**

This protocol is cost-effective and sensitive to budget limitations and the potential economic and logistical strains placed on the national Ministries of Health. Here we present a high resolution map of disease prevalence levels, and treatment regimens are recommended.

## Author Summary

Historical data indicates Namibia, particularly northern Namibia, as endemic for geohelminths and schistosomiasis, albeit to a lower extent than other areas in Sub-Saharan Africa. The National Ministry of Health and Social Services, with extensive backing from other governmental and non-governmental organizations, investigated the extent of the problem in preparation for deployment of a control programme. Using a cost-effective strategy, a new generation protocol was developed for mapping these important neglected tropical diseases, bolstering field-standard microscopy results with those from commercially available rapid diagnostic tests. The protocol used increased the mapping coverage (one in every four schools mapped) with minimal added cost. Results from our surveys, which included 17 896 school-going children (3–19 years of age), identified hotspots of transmission for schistosomiasis and geohelminths, particularly hookworm infection, and allowed the recommendation of constituency- and region- specific treatment regimens, as well as improvements to water, sanitation and hygiene. Furthermore, we identified high levels of intestinal morbidity, believed to be associated with hookworm and *Hymenolepis nana* infections, and lower levels of urogenital pathology, associated with *Schistosoma haematobium* infection. A cost-effectiveness analysis is also presented in addition to reporting the diagnostic accuracy of the rapid diagnostic tests employed.

## Introduction

Namibia has recently established a national programme for the integrated control of neglected tropical diseases (NTDs) with support from The END Fund. The programme's first objective was to gather detailed information on the prevalence and distribution (mapping) of schistosomiasis (both intestinal and urogenital) and soil-transmitted helminths (STH). Although other NTDs are endemic in Namibia, of the five eligible for preventive chemotherapy (PCT), only schistosomiasis and STH infections are believed to be prevalent [1]. Lymphatic filariasis and onchocerciasis have never been identified at the community- or health facility-level [2,3], and for trachoma, although indicated by predictive mapping [4,5], no epidemiological confirmation has been reported [6].

According to WHO NTD maps, Namibia is indicated to have a prevalence of schistosomiasis below 10% [7]. Historical data from the northern regions report heterogeneity in infection distribution with high transmission areas reportedly reaching 95% prevalence [2,8–11]. The presence of both *Schistosoma haematobium* and *S*. *mansoni* in Namibia has been confirmed with *S*. *mansoni* distribution confined to the Kavango and Kwando rivers due to lack of *Biomphalaria* spp. snails in any other areas [11]. According to historical data, all three major STH infections (hookworm, *Ascaris* and *Trichuris*) have been confirmed in northern Namibia, with hookworm reported as dominant [2,9,11–13]. *Ascaris* and *Trichuris* infections are largely absent with levels usually below 1% (with few exceptions among the San people) due to the arid climatic conditions [12] and behavioural factors that exist among sparse host populations [13] (reviewed by [1]).

Although there is significant historical data indicating the presence of schistosomiasis and STHs in Namibia, no large-scale systematic study capable of guiding a drug administration campaign has been conducted in the past two decades. Furthermore, for the past 17 years, deworming tablets (albendazole or mebendazole) have been regularly distributed to children during national immunization days plus (polio, vitamin A and deworming) [14], which means prevalence levels have likely changed. Therefore, a new integrated rapid mapping protocol was developed to define areas that required different interventions, to estimate drug requirements, to target mass drug administration (MDA) of PCT to at-risk populations, to select appropriate control measures and to determine frequency of interventions. This newly developed protocol employed a sample size calculation method using mapping resolution, determined optimal treatment frequency among pre-school aged children, used rapid diagnostic tests (RDTs) for the diagnosis of schistosomiasis and utilized haematuria (both visual and microscopic) and bowel morbidity marker to better evaluate future interventions.

## Methods

### Ethical statement, recruitment and treatment

This protocol was approved by the Liverpool School of Tropical Medicine (ref: LSTM 12.37) and was registered as a project within the Ministry of Health and Social Services (MoHSS) of Namibia. The protocol was reviewed by the MoHSS and implemented following their recommendations. Before selection, school principals received an information leaflet (in local languages) detailing the objectives and procedures of this study. The study was fully explained by a MoHSS officer to those who chose to participate. Before enrolment, informed consent in writing was given by the school principals. After collection of samples, all children were offered a standard doses of PZQ, 40mg/kg (CIPLA, Mumbai, India), and albendazole, or ALB, single 200mg tablet (GSK, Uxbridge, UK), following WHO guidelines [15]. All treatment was supervised and confirmed by a MoHSS officer. Participation in these surveys was voluntary. If a child refused to take part in the study, or their parents decided to opt-out, then no treatment was given at the time of the study.

### Study site

**Fig 1:**
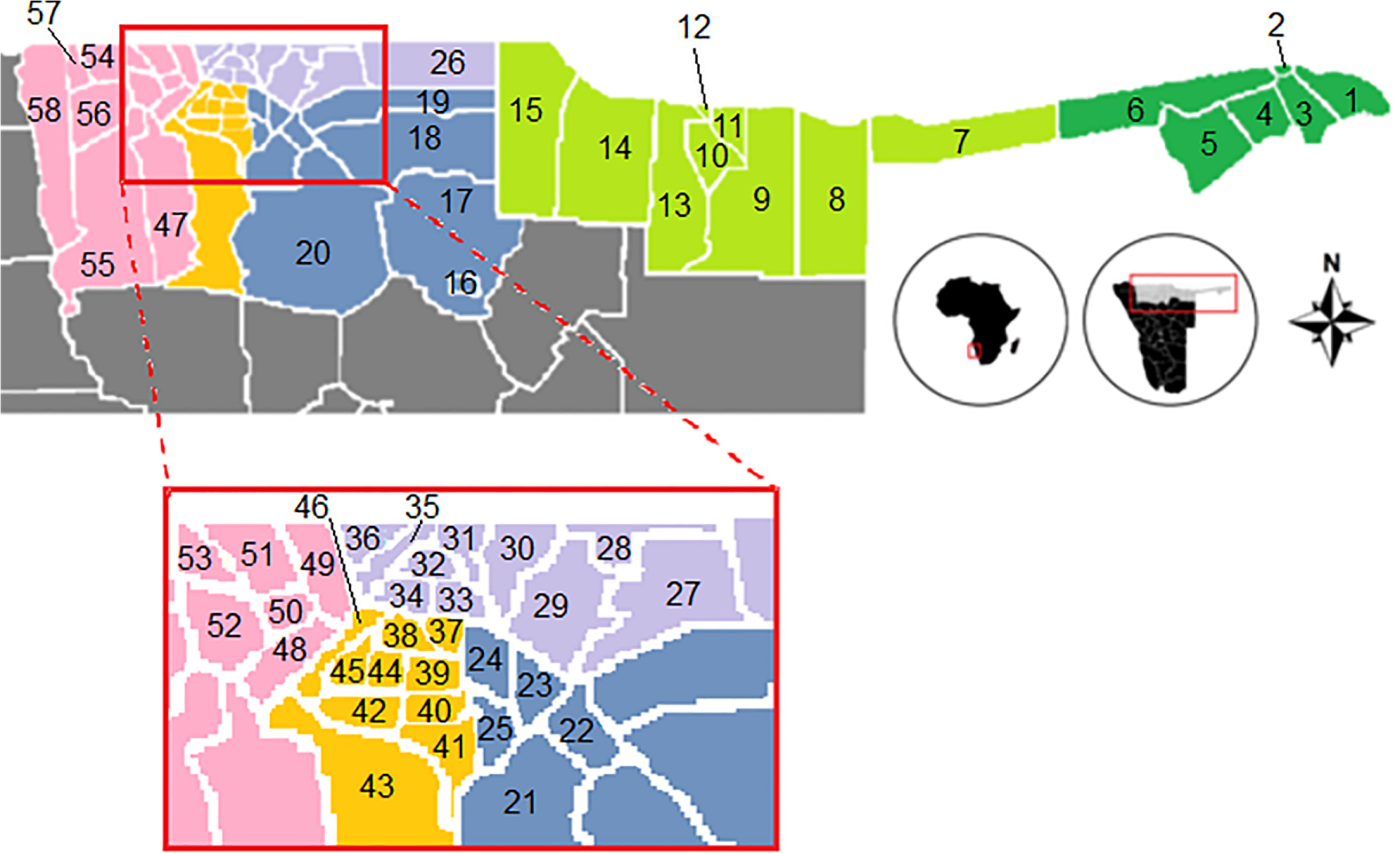
Map of Northern Namibia’s governmental regions and constituencies. Regions are color-coded, with dark green for Caprivi, light green for Kavango (Phase 1), blue for Oshikoto, purple for Ohangwena, orange for Oshana and pink for Omusati (Phase 2). Constituencies are number-coded: 1 –Kabe, 2 –Katima Mulilo Rural, 3 –Katima Mulilo Urban, 4 –Sibinda, 5 –Linyati, 6 –Kongola, 7 –Mukwe, 8 –Ndiyona, 9 –Mashare, 10 –Rundu Rural West, 11 –Rundu Rural East, 12 –Rundu urban, 13 –Kapako, 14 –Kahenge, 15 –Mpungu, 16 –Tsumeb, 17 –Guinas, 18 –Eengondi, 19 –Okankolo, 20 –Omuthiyagwiipundi, 21 –Omuntele, 22 –Onyaanya, 23 –Onayena, 24 –Oniipa, 25 –Olukonda, 26 –Okongo, 27 –Epembe, 28 –Omundaungilo, 29 –Eenhana, 30 –Ondobe, 31 –Oshikango, 32 –Ohangwena, 33 –Omulonga, 34 –Endola, 35 –Engela, 36 –Ongenga, 37 –Okaku, 38 –Ongwediva, 39 –Ondangwa, 40 –Uukwiyo, 41 –Okatyali, 42 –Opundja, 43 –Uuvudhiya, 44 –Oshakati East, 45 –Oshakati West, 46 –Okatana, 47 –Otamanzi, 48 –Elim, 49 –Etayi, 50 –Oshikuku, 51 –Okalongo, 52 –Ogongo, 53 –Anamulenge, 54 –Outapi, 55 –Okahao, 56 –Tsandi, 57 –Onesi, 58 –Ruacana. Rest of the country (in grey) is not included in this report.

Namibia is sparsely populated, rivalled only by Mongolia with low population density, with many Regions (equal to provinces) larger than 100,000 km^2^ (bigger than Scotland and Wales together). Because of the logistics involved and the innovative approach envisioned, the implementing partners divided the country into three phases according to epidemiological and demographic data in order to maximize cost-effectiveness: Phase 1 covered the wettest area of the country, Caprivi and Kavango regions; Phase 2 covered the northern most populated regions of Omusati, Oshana, Oshikoto and Ohangwena [16]; and Phase 3 covered the largely arid regions of Kunene, Otjozondjupa, Khomas, Erongo, Omaheke, Hardap and Karas (Fig 1). Due to a lack of historical/hospital data records for local transmission of STH and schistosomiasis infections in Phase 3 regions [1], the mapping was considered a low priority and was not included in this publication. Caprivi has recently been renamed Zambezi region, and Kavango region has been divided into Kavango West and Kavango East [17].

Namibian governmental regions further subdivide into constituencies, the implementation units for health and education (Fig 1).

### Mapping coverage

According to data provided by the Government of Namibia (National census 2011[18]) and the Ministry of Education (MinEd, 2012/2013), the two mapping phases cover around 18% (148 116 km^2^) of Namibia's geographical area, 78% of all schools in the country (27% for Phase 1 and 51% for Phase 2) and 55% of the 2.1 million population.[19] Phase 1 field-work was conducted in November 2012 and Phase 2 field-work was conducted in November 2013.

### Protocol

#### Mapping resolution

WHO guidelines for the epidemiological assessment of helminths, in particular schistosomiasis, are not very precise. Most WHO documents do not mention a detailed methodology while others use broad terms such as "5–10 schools per ecological zone", "a sample of schoolchildren should be selected in each of the ecological zones of the country", or even "use data available in-country" [15,20,21]. To optimize current guidelines, this protocol was developed specifically for Namibia. Instead of relying on ecological zones or implementation units (which can vary widely in Africa from 10 000 km^2^ to 100 000 km^2^), we introduced the term "mapping resolution" in the protocol developed. Just as in electronic images, the higher the resolution, the higher the detail. Since schistosomiasis is heterogeneously distributed, highly dependent on presence of freshwater and intermediate hosts, and STH infections are more homogeneous distributed, only dependent on soil and climatic factors,[15] we argue that the first requires a higher resolution map compared to the latter.

#### Younger school-aged children

As an addition to standard school-based mapping surveys, we opted to include a cohort of 5–7 year olds, alongside the usual cohort of 10–15 year olds. The older cohort was used to determine the need for treatment at the school level following WHO recommendations [21], and the younger cohort was used as a proxy to determine the need for treatment among pre-school aged children. There is sufficient evidence that this younger age class is at risk of infection and disease [22], and so it was decided to assess status of infection at school entry level, effectively doubling our sample size.

#### Rapid diagnostic tests

Although RDTs are widely used in research and mapping of some NTDs [21,23], there is a gap regarding protocol optimization for schistosomiasis.[24] With this protocol, we introduced the concept of rapid diagnostic teams working alongside standard mapping teams. These rapid diagnostic teams used RDTs only, assessing prevalence of *S*. *haematobium* and *S*. *mansoni* infections. The microscopy teams used RDTs alongside standard microscopy to assess diagnostic performance.

### Sample size calculation

For the sample size calculation for schistosomiasis, we relied on historical data[25] and Epi Info 7 software (CDC, Atlanta, USA). According to Ministry of Education data, there were 408 804 children enrolled in primary schools in Namibia in 2011. Given the annual population growth rate of 1.8% our target student population was estimated at 416 162 children in 2012. Assuming a background prevalence of infection of 11% in Namibia,[26] a precision of ±5%, confidence levels of 99.9%, a design effect of 2.5, correcting for a cluster design assuming the existence of 1497 clusters (no. of schools), the resulting sample size was 11 220 school-aged children to be mapped in Namibia (after rounding). For Phases 1 and 2, the resulting sample estimation was 9 090 school-aged children. This number was then used for mapping resolution. After weighing in country-specific characteristics such as size, distribution of people and schools, and travel distances, the most reasonable, yet scientifically sound mapping resolution was to visit one in every four schools– 303 schools of the 1 226 in Phases 1 and 2, testing 30 children per school. Since a cohort of younger school-aged children in our study was required, our effective sample size was doubled to 60 students per school, meaning that our target sample size was 18 180 children from 303 schools for Phases 1 and 2.

For STH infections, sample size calculation was determined according to WHO guidelines [21]. Given the budget and the eight ecological zones in Namibia[19], we opted to visit 9 schools per ecological zone. The resulting mapping resolution for microscopy was one in every twenty schools. This meant that of the 303 schools to be mapped using rapid diagnostic tests in Phases 1 and 2, 61 schools were targeted for mapping using standard microscopy techniques for schistosomiasis and STH infections alongside the rapid diagnostic tests. Our target number of children to be mapped using standard microscopy was 1830 older school-aged children (10–14 year olds) and 1 830 younger school-aged children (5–7 year olds) (3 660 of the 18 180 children to be mapped by RDTs)

These mapping resolutions were tailor-made for Namibia and should be adapted if applying this protocol in smaller more densely populated countries (See S1 Table for a summary of sample size targets).

### Inclusion and exclusion criteria

Schools were randomly selected from a list of all primary schools (private and public) in the selected geographical area (the sampling frame). After selection, the schools were plotted on a map and adjustments were made to reduce urban bias thus ensuring geographical representation (structured random sampling). Students were selected based on systematic random sampling from a sampling frame generated on the day of the study, independent of age and sex. Students who did not wish to participate in the study were excluded from the sampling frame.

### Data collection

In each region, teams worked simultaneously in the field: two (sometimes three in larger regions) carried out mapping by microscopy and RDTs, visiting up to one school per day each. The other two teams, composed of 8–10 rapid diagnostic test technicians, visited up to ten schools per day (one per technician). Paper data entry forms were used to collect information from the field. Each form consisted of general school information (region, constituency, school name, GPS position, name of principal and contact details, total number of classes taught, total number of teachers available and total number of children, boys and girls, enrolled) and a suit of questions directed at the school principal (presence of latrines for students and their working condition, presence of potable water and source, history of deworming campaigns and if teacher was willing to treat children in the future).

### Parasitological diagnosis

#### Microscopy

Parasitological diagnosis of *S*. *mansoni* and STH infections was performed using a single Kato-Katz (KK) thick smear prepared from a single day stool samples (41.7mg of stool per smear) [27]. Parasitological diagnosis of *S*. *haematobium* was performed on each sample of mid-morning urine, where 10ml were syringed through a Millipore filter (12μm polycarbonate filter) for detection of eggs [28]. Results were expressed as eggs per gram of faeces (epg) for *S*. *mansoni* and STH infections, and in number of eggs per 10ml of urine for *S*. *haematobium*, and infection intensities were categorised according to WHO guidelines [21]. Microscopy was conducted by Ministry of Health technicians and supervised by a senior technician for quality control. Only 3 659 (from 61 schools) were also tested using standard microscopy techniques.

#### Rapid diagnostic tests for infection

Rapid diagnosis of *S*. *mansoni* was performed using a single urine sample from each student for testing the presence of schistosome circulating cathodic antigen (CCA) with a commercially available immuno-chromatographic dipstick (Rapid Medical Diagnostics, Pretoria, RSA) [29]. Trace results were considered negative due to results from ROC analysis presented here. Each sample was also tested for microhaematuria using the Hemastix reagent strip (Bayer, UK), a known proxy for *S*. *haematobium* infection [30]. A total of 17 896 children from 299 schools were tested using RDTs.

For prevalence assessment purposes, a child was considered positive for infection either if positive by rapid diagnostic test or microscopy.

### Morbidity markers

Visual blood in urine (macrohaematuria) was assessed in every urine sample collected by trained technicians, as an indicator of a *S*. *haematobium*-related urogenital pathology [21]. Faecal occult blood (FOB) rapid diagnostic tests were performed on all stool samples provided to assess the presence of microscopic levels of blood in stool. This allowed estimation of bowel morbidity, proxy for disease due to infection with *S*. *mansoni* and STH infections [31,32].

### Statistical analysis

Data were collected using pro-forma data sheets in the field, and then entered using EpiData (The EpiData Association, Odense, Denmark). The data thus collated were analysed using the R statistical package v. 2·10·1 (The R Foundation for Statistical Computing, Vienna, Austria), Microsoft Excel spreadsheet software, ArcGIS (Release 10.1: Environmental Systems Research Institute, Redlands, CA), and MedCalc software v. 14.8.1.0 (MedCalc, Ostend, Belgium). For percentage values, 95% confidence intervals (CI_95_) were estimated using the exact method [33]. Prevalence comparisons were performed using (one-tailed) Fisher's exact modification of the 2 × 2 chi-squared test [34]. For infection intensity values, the geometric mean of Williams, GM_W_, was chosen as the measure of central tendency due to the typical over-dispersion present in this type of data [35]. Schools, constituencies and regions were characterised according to infection prevalence levels, and treatment regimens are recommended in this article following WHO guidelines [36].

Logistic regression was carried out to ascertain associations between morbidity indicators and infections. Since individuals from the same school are more likely to experience the same conditions, we accounted for intra-correlation in the data using a generalized linear mixed model with multivariate normal random effects (the random-effects of school in our case), with penalized quasi-likelihood using the function glmmPQL in R [37]. All models controlled for sex and age. For each variable, adjusted odds ratio (OR) and *P*-values were calculated, and a *P*-value < 0.05 was considered indicative of statistical significance.

### School-level analysis and geospatial analysis

First, the effects of water (access to water or no access to water), plus sanitation (access to good quality latrines, access to bad quality latrines or no access to latrines) on schistosomiasis prevalence was assessed using a logistic regression modelling approach.

Digitised map data relating to freshwater sources were used to determine the distance between potential transmission sites and schools in kilometres [38]. Further, remotely sensed data relating to elevation, slope [39], total annual rainfall, average maximum temperature [40] and Normalised Difference Vegetation Index (NDVI) [41] were extracted at the locations of each of the surveyed schools. A multiple logistic regression model was fitted to the prevalence data. All variables were included in the model (elevation, slope, maximum temperature, total annual rainfall, NDVI, Square Root Distance to Freshwater, water and sanitation), with the square root of the distance to the nearest freshwater source being used due to this measure being highly positively skewed. Model selection was undertaken using a backwards stepwise selection approach based on minimising Akaike Information Criterion (AIC) [42]. Due to concerns with the lack of variability in three of the eight variables, a second model was also fitted which excluded these three (temperature, elevation, slope). The fit of these models was assessed by calculating the sensitivity and specificity of the resulting fitted values with respect to a range of prevalence thresholds.

The global Moran’s I statistic was calculated to test for evidence of spatial clustering of prevalence estimates in surveyed schools using inverse distance weights [43]. Further to this, evidence of local spatial clustering was assessed using Anselin local Moran’s I statistic. In order to determine whether any observed spatial clustering was a result of schools sharing similar characteristics, these statistics were recalculated using the residuals (observed-fitted values) obtained from the fitted logistic regression model. Clusters of high residuals would indicate that there may be further unmeasured variables influencing the spatial distribution of the disease.

### Diagnostic performance

Diagnostic performance of both CCA and Hemastix as diagnostic tests for *S*. *mansoni* and *S*. *haematobium*, respectively, was established using light microscopy (KK technique and urine filtration, respectively) as ‘field-standards’. Sensitivity (SS), specificity (SP), positive predictive value (PPV) and negative predictive value (NPV) were calculated [44]. The diagnostic performance of each test was calculated using all children as a single population.

Receiver operating characteristics (ROC) analyses were also performed [45], plotting the true positive rate (Sensitivity) in function of the false positive rate (100-Specificity). Calculation of standard error (SE) of the area under the curve (AUC) was performed according to DeLong and colleagues [46]. For ROC analysis, microscopy methods were considered the ‘field-standard’ (as a binomial variable) against which results from haematuria (as a continuous variable whereby: negative = 0, trace non-haemolysed = 1, trace haemolysed = 2, + = 3, ++ = 4, +++ = 5) and the urine CCA test (as a continuous variable whereby: negative = 0, trace = 1, + = 2, ++ = 3, +++ = 4) were measured for performance.

## Results

### Study population

Of a targeted 18 180 children from 303 schools, 17 896 children from 299 schools (98.4%) were tested; 58 schools in Ohangwena, 65 schools in Omusati, 32 schools in Oshana, 45 schools in Oshikoto, 23 schools in Caprivi and 76 schools in Kavango (Table 1 and S2 Table). Age of children surveyed ranged between 3 and 19 years of age, mean age 9.6. There was an equal proportion of boys and girls in the survey and an equal proportion of young (3–7 year olds) and older (8–19 year olds) school-aged children. All children were recruited from primary schools. Of the 17 896 RDT-tested children, 3 659 (61 schools) were also randomly selected to be tested using standard microscopy techniques and faecal morbidity marker.

**Table 1:** Prevalence of schistosomiasis per region, according to rapid diagnostic tests and microscopy. *S*. *haematobium* is detected by both dipstick and urine filtration, *S*. *mansoni* is detected by single CCA test and single stool Kato-Katz; *Schistosoma* spp. prevalence is determined by any diagnostic method.

Constituency	N schools	N students	Hemastix		CCA		*S*. *haematobium*		*S*. *mansoni*		*Schistosoma* spp.	
			%	CI_95_	%	CI_95_	%	CI_95_	%	CI_95_	%	CI_95_
Ohangwena	58	3479	3·0	2·4–3·6	1.2	0.9–1.7	3.0	2.4–3.6	1.2	0·9–1.7	4.1	3.5–4.9
Omusati	65	3880	4·1	3·5–4·8	1.0	0.7–1.3	4.1	3.5–4.7	1.0	0.7–1.4	5.0	4.4–5.8
Oshana	32	1919	5·6	4·6–6·7	1.3	0.9–1.9	5.6	4.6–6.7	1.3	0.8–1.9	6.7	5.6–7.9
Oshikoto	45	2700	2·9	2·3–3·6	0.7	0.4–1.1	2.9	2.3–3.6	0.7	0.4–1.1	3.4	2.8–4.2
Caprivi	23	1380	6·5	5·3–8·0	10.2	8.7–11.9	6.7	5.5–8.2	10.2	8.7–11.9	16.1	14.2–18.1
Kavango	76	4538	7·9	7·1–8·7	11.4	10.5–12.4	8.1	7.3–8.9	11.5	10.5–12.4	18.2	17.1–19.3
TOTAL	299	17896	5·0	4·7–5·3	4.4	4.1–4.7	5.1	4.7–5.4	4.4	4.1–4.7	9.0	8.6–9.4

### Questionnaire

Overall, 92% of schools had latrines, but only 75% had latrines in good working condition, and 88% of schools had a safe water source. In the schools with safe water source, 65% had access to tap water and 35% had access to borehole water. We were informed that Namibia had implemented albendazole distribution during national immunization days in 2012/2013 targeting school-aged children. Data gathered during the questionnaire informs that coverage of this campaign was minimal with only 23% of school having received treatment in 2012/2013 and 27% of schools having received treatment in the recent past. For graphical representation please see S1 Fig.

### Schistosomiasis

Overall, schistosomiasis prevalence in the surveyed areas was 9.0% (Table 1). The lowest prevalence was registered in Oshikoto region (3.4%) and the highest in Kavango (18.2%). In fact, Caprivi and Kavango, the two regions mapped during Phase 1, had the highest recorded prevalence levels. The constituency with the highest recorded prevalence of schistosomiasis was Kongola in Caprivi with levels reaching 48.3% of which 39.2% were positive for *S*. *mansoni*, 9.2% were positive for *S*. *haematobium* and 0.1% were co-infections. The most prevalent form of schistosomiasis was urogenital (5.1%), closely followed by the intestinal form (4.4%).

**Fig 2:**
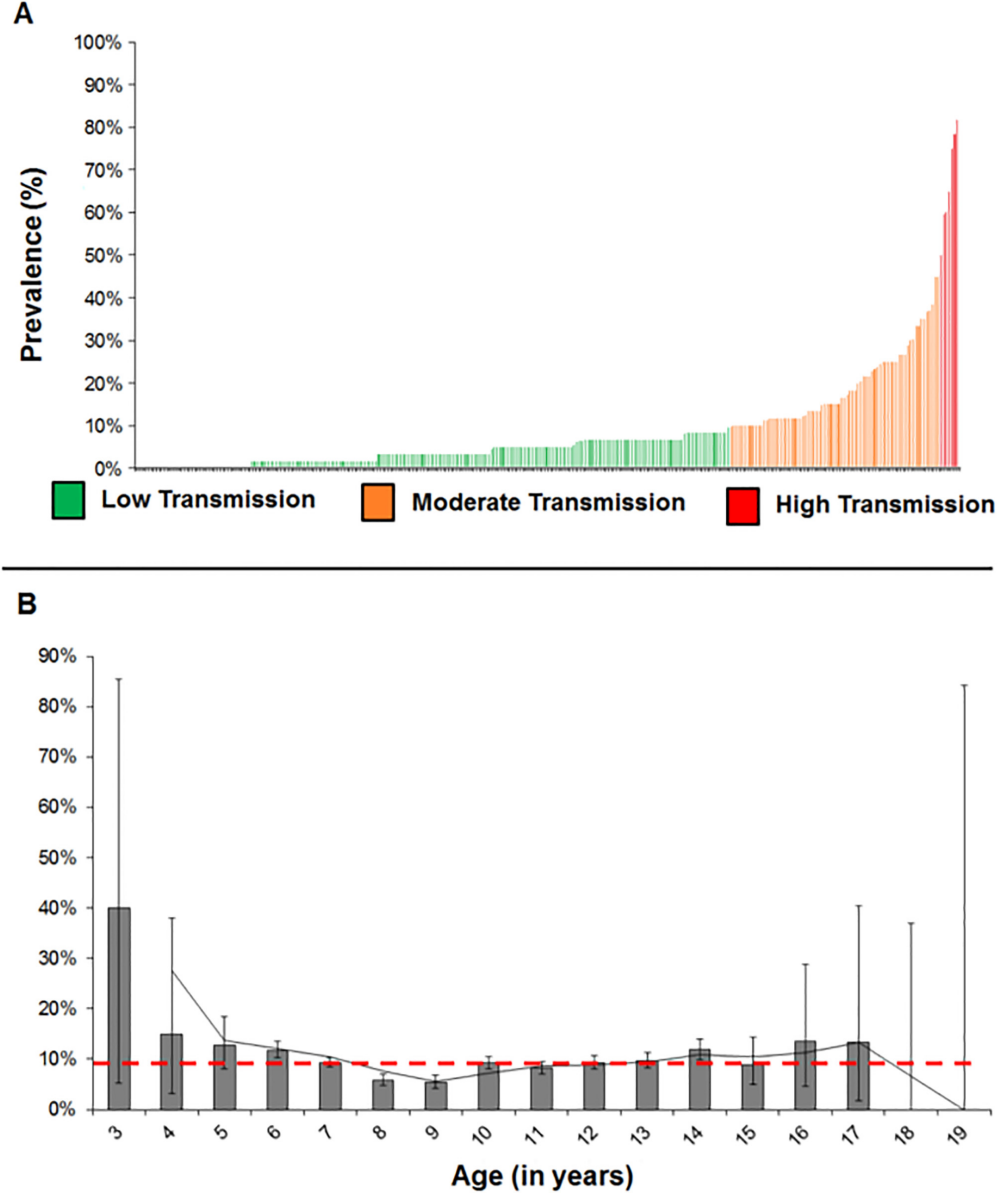
Dynamics of schistosomiasis in northern Namibia (299 schools, 17 896 children ages between 3 and 19). **A)** Binomial distribution of schistosomiasis in schools, color-coded according to transmission following WHO guidelines [36]: low transmission is 0.1–9.9% prevalence, moderate transmission is 10–49.9% prevalence and high transmission is prevalence level equal or above 50%. **B)** Age-frequency distribution of schistosomiasis, with red dashed line indicating the overall average of 9.0%, and vertical confidence intervals. For more information see Appendix 1.

Of the 299 schools surveyed, seven were considered of high schistosomiasis transmission (above 50.0% prevalence), with the highest being 81.7% in Katwitwi Primary School, Mpungu Constituency, Kavango Region (76.6% *S*. *mansoni*, 33.3% *S*. *haematobium* and 28.2% co-infections). Furthermore, a total of 75 schools registered moderate transmission (10.0–49.9%), 175 registered low transmission (0.1–9.9%) and 42 registered no transmission at all (Fig 2A).

Age-frequency distribution of schistosomiasis in this part of Namibia was not binomially distributed (Fig 2B). Most age-frequency confidence intervals overlapped the overall mean of 9%, meaning they were not significantly different, except for ages 6 and 14 (both at 12%) and ages 8 and 9 (both at 6% and 5%, respectively).

#### Schistosomiasis and the environment

In a multivariate model, both latrine accessibility/quality and water accessibility influenced schistosomiasis risk. Compared with those having good quality latrines, those in schools with bad quality latrines were 1.2 times (CI_95_ 1.0–1.4, *P* = 0.008) more likely to be infected with schistosomiasis and those with no latrines were 2.0 times (CI_95_ 1.7–2.4, *P*<0.001) more at risk. Similarly, those attending schools where there was no access to drinking water sources were 1.5 times (CI_95_ 1.3–1.8, *P*<0.001) more likely to be infected with schistosomiasis compared with those with water access. Upon separating the data into *S*. *mansoni* and *S*. *haematobium*, the risk associated with poor water and sanitation was much greater for *S*. *mansoni* than *S*. *haematobium*. For example, those in schools with no latrines were 2.7 times (CI_95_ 2.3–3.3, P<0.001) more at risk of *S*. *mansoni* infection, and only 1.25 times (CI_95_ 1.0–1.6, P = 0.065) more at risk of *S*. *haematobium* infection.

**Fig 3:**
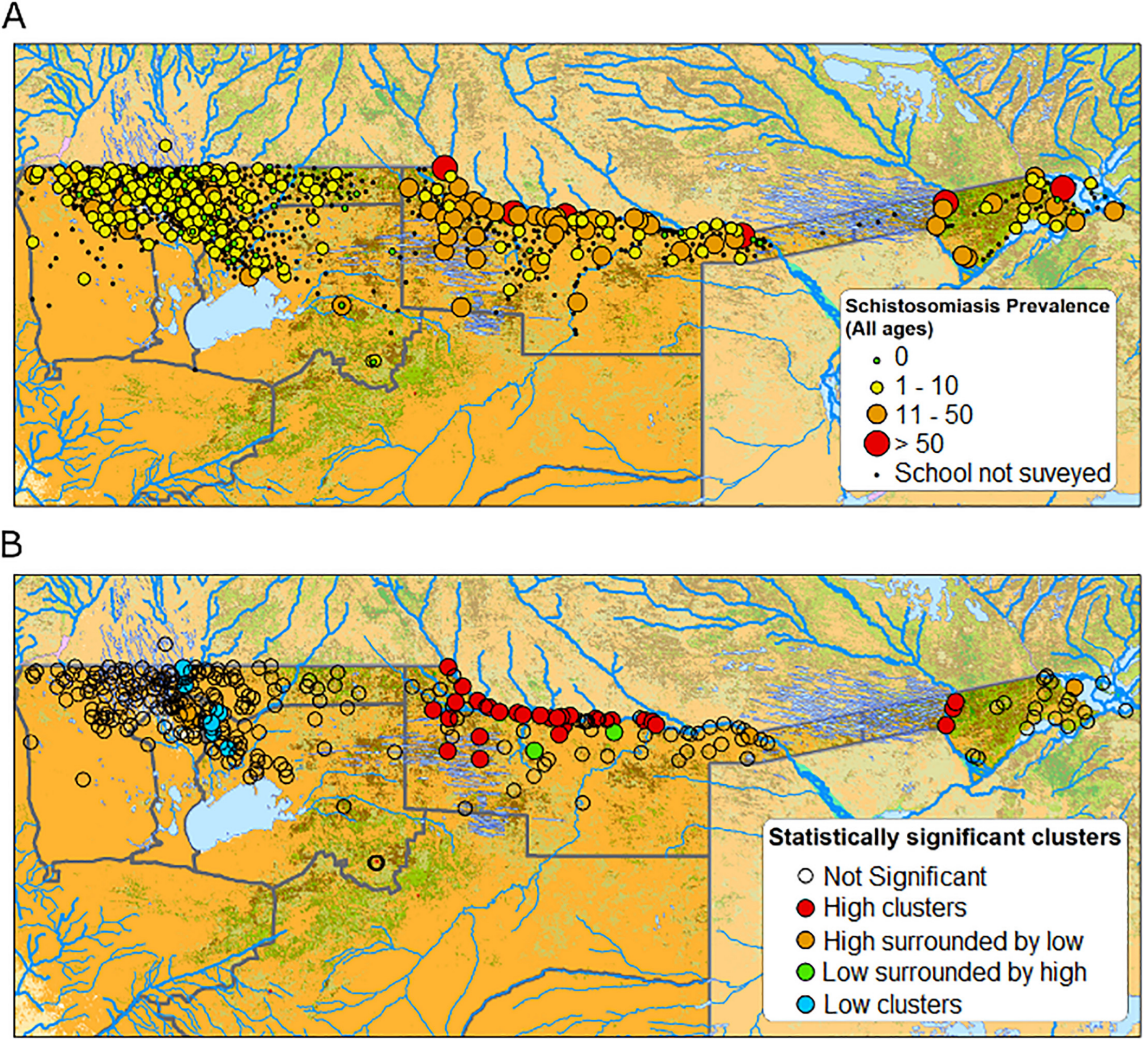
Schistosomiasis prevalence in the study area. **A)** Distribution of surveyed schools; **B)** Local Moran’s I results for schistosomiasis prevalence.

Results from the stepwise logistic regression of the two models with environmental variables, water and sanitation are presented in Table 2. Model 1 considers all significant potential environmental predictors, whereas Model 2 excluded altitude, slope and temperature. While significant in Model 1, water access was not statistically significant in Model 2. Model 1, containing all six environmental variables plus water and sanitation, had a reasonably high sensitivity (Fig 3) and successfully identified 70% of schools that had a prevalence level above 10%. This predictive ability of Model 1, however, rapidly declined as the threshold increased. Between the 10% and 20% prevalence level, the proportion of schools exceeding this threshold dropped dramatically from around two thirds to approximately 10%. For graphical representation please see S2 Fig).

**Table 2:** Results of the multiple logistic regression using environmental variables plus water and sanitation. Model 1 includes all eight variables, while Model 2 selection started with only five variables (Elevation, Slope and Maximum Temperature were removed). NDVI stands for Normalised Difference Vegetation Index.

	Model 1			Model 2		
Variable	OR	95% CI	*P*-value	OR	95% CI	*P*-value
Elevation	0.996	0.995–0.997	<0.0001	-	-	-
Slope (degrees)	1.228	1.140–1.322	<0.0001	-	-	-
Maximum Temperature	0.902	0.887–0.918	<0.0001	-	-	-
Total Annual Rainfall	1.007	1.006–1.008	<0.0001	1.004	1.003–1.005	<0.0001
NDVI	2.008	1.014–3.972	0.0453	8.562	4.597–15.948	<0.0001
Square Root Distance to Freshwater	0.845	0.814–0.876	<0.0001	0.841	0.812–0.872	<0.0001
Latrines						
Good quality						
Bad quality	1.249	1.091–1.426	0.0011	1.227	1.075–1.397	0.0022
Not available	1.512	1.284–1.774	<0.0001	1.617	1.377–1.892	<0.0001
Water access						
Available						
Not available	0.779	0.666–0.908	0.0016	-	-	-

#### Mapping schistosomiasis

The median schistosomiasis prevalence across the 299 schools surveyed was 5.00 with an inter-quartile range of 1.67–10.00 (Fig 3A). At the regional level, prevalence in Caprivi and Kavango was much greater than that observed in the remaining four regions. Fig 3A also highlights the high level of heterogeneity in prevalence in Caprivi and Kavango, whereas prevalence rarely exceeds 10% in the remaining four regions. The global Moran’s I statistic was non-significant for school-level prevalence with a p-value of 0.29, thus reflecting the overall heterogeneity of prevalence values across the study area. The Anselin Local Moran’s I statistic, however, highlighted a statistically significant cluster of high values in northern Kavango (Fig 3B). After adjusting for the effects of water and sanitation, plus environmental risk factors on prevalence (Model 1), further spatial clustering in the residuals was identified in northern Kavango using Anselin Local Moran’s I statistic, indicating that there were unmeasured risk factors influencing prevalence in this area.

### Soil transmitted helminths

**Fig 4:**
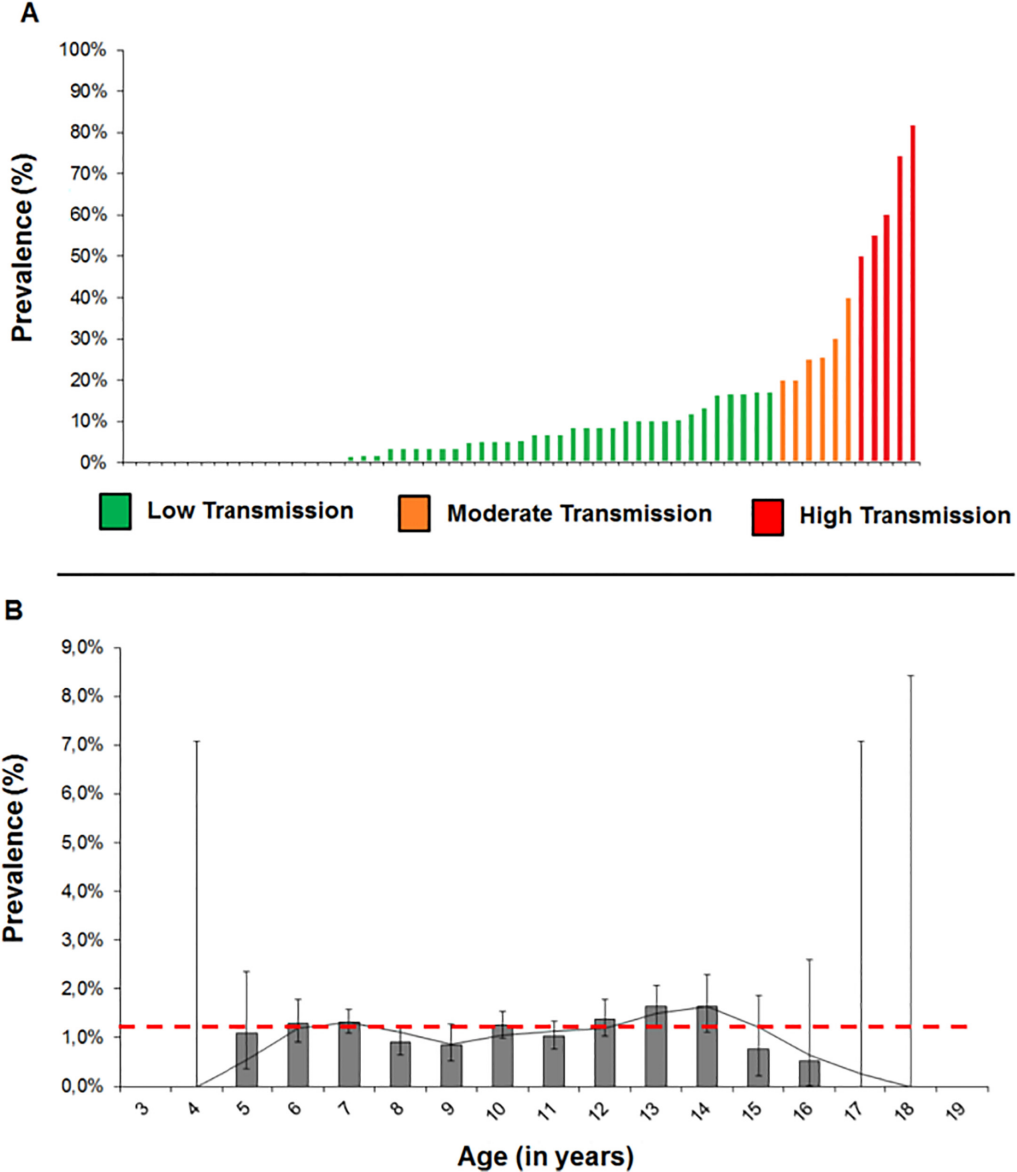
Dynamics of hookworms in northern Namibia (61 schools and 3 659 children ages 4 to 18). **A)** Distribution of schistosomiasis in schools, color-coded according to transmission following WHO guidelines:[36] low transmission is 0·1–19·9% prevalence, moderate transmission is 20·0–49·9% prevalence and high transmission is prevalence level equal or above 50%. **B)** Age-frequency distribution of schistosomiasis, with red dashed line indicating the overall average of 12·2%, and horizontal confidence intervals. For more information see Appendix 1.

Of the three most common STH infections, only hookworm infection was present in the study population with overall prevalence of 12.2% and few high intensity infections (0.1%) (Table 3). The lowest prevalence was registered in Omusati region (2.1%) and the highest in Kavango (28.5%). The constituency with the highest recorded prevalence of hookworms (63.9%) was Mpungu in Kavango region (Fig 4A). Only one case of *A*. *lumbricoides* infection was detected (144 epg) in a nine year old boy from Ohangwena region. *Trichuris* cases were not found.

**Table 3:** Prevalence of hookworm and *Hymenolepis nana* infections and intestinal and urogenital morbidity. Hookworms and other soil-transmitted helminths were detected solely by microscopy (Kato-Katz technique), intestinal morbidity by the rapid diagnostic test faecal occult blood (FOB), and urogenital morbidity represented to visual blood in urine (VBU), or macrohaematuria.

Constituency	N	N	Hookworm		*H*. *nana*		FOB		VBU	
	schools	students	%	CI_95_	%	CI_95_	%	CI_95_	%	CI_95_
Ohangwena	12	718	16.0	13.4–18.9	1.1	0.5–2.2	17.8	15.1–20.8	0.2	0.1–0.4
Omusati	14	840	2.1	1.3–3.4	1.7	0.9–2.8	24.8	21.9–27.8	0.3	0.2–0.5
Oshana	6	359	4.7	2.8–7.5	1.4	0.5–3.2	17.0	13.3–21.3	0.3	0.1–0.7
Oshikoto	9	541	5.4	3.6–7.6	0.7	0.2–1.9	25.1	21.5–29.0	0.4	0.2–0.7
Caprivi	5	298	3.7	1.9–6.5	1.0	0.2–2.9	2.7	1.2–5.2	0.4	0.1–0.8
Kavango	15	903	28.5	25.5–31.5	4.9	3.6–6.5	3.3	2.3–4.7	0.3	0.2–0.5
TOTAL	61	3659	12.2	11.2–13.3	2.1	1.7–2.7	15.6	14.4–16.8	0.3	0.2–0.4

Age-frequency distribution of hookworms in this part of Namibia was not binomially distributed nor did frequency increase with age (Fig 4B). Most age-frequency confidence intervals overlap the overall mean of 12.2%, meaning they are not significantly different, except for ages 8, 9 and 11 (prevalence levels of 9.1%, 8.4% and 10.2%, respectively).

Apart from the common STH infections, we also registered significant levels of *Hymenolepis nana* infections with levels reaching 4.9% in Kavango region (2.1% prevalence overall) (Table 3)

### Morbidity markers

The overall prevalence of intestinal morbidity as detected by the rapid diagnostic test FOB was 15.6% (N = 3 659), with levels ranging from 2.7% in Caprivi and 25.1% in Oshikoto. There was a significant association between intestinal morbidity as detected by the FOB test and hookworm (OR = 1.9, CI_95_ 1.4–2.6, P<0.001) and *H*. *nana* infections (OR = 1.9, CI_95_ 1.1–3.4, P = 0.031). In the same model, age and sex were also significantly associated with FOB, whereby older children have a higher likelihood of being positive (OR = 1.07 for every additional year of age, CI_95_ 1.03–1.11, P<0.001) and girls were more likely to be positive than boys (OR = 1.3, CI_95_ 1.1–1.5, P = 0.006).

The overall prevalence of visual blood in urine, a proxy for urogenital morbidity, was very low, reaching only 0.3% (N = 17 896), with maximum recorded prevalence of 0.4% in Oshikoto and Caprivi. Urogenital pathology was significantly associated with *S*. *haematobium* infection (OR = 13.2, CI_95_ 10.7–16.2%, *P*<0.0001), as detected by RDT and light microscopy. In the same model, age and sex were also significantly associated with visual blood in urine whereby older children have a higher likelihood of being positive (OR = 1.15 for every additional year of age, CI_95_ 1.11–1.19, P<0.001) and girls were less likely to be positive than boys (OR = 0.7, CI_95_ 0.6–0.9, P = 0.006).

### Diagnostic accuracy

When considering light microscopy (Kato Katz technique and urine filtration) as the ‘field-standard’, the overall sensitivity of the urine CCA was 81.8% (CI_95_ 59.7–94.8%) and the haematuria rapid test (Hemastix) was 83.2% (CI_95_ 75.0–89.6%). The overall specificity of the urine CCA was 98.1% (CI_95_ 97.6–98.5%) and of the Hemastix was 95.9% (CI_95_ 95.1–96.5%). Because light microscopy was considered the ‘field-standard’, diagnostic performance analysis, including ROC analysis, was conducted on a sample size of 3 659 children. In this subset of our data, both *S*. *mansoni* and *S*. *haematobium* infections were rare with prevalence levels reaching 0.6% and 3.1%, respectively.

Graphical representations of the ROC curves, AUC and respective standard errors of the urine CCA test and the Hemastix test are presented in S3 Fig. The AUC for the CCA test was 0.941 (CI_95_ 0.933–0.948, *P*<0.0001), with a Younden J index of 0.7992. Of note, the ROC analysis showed that in such a low transmission setting (prevalence below 10%), the urine CCA test performed better if the observer considered trace results as negative cases. If trace results were considered as positive cases, it would have rendered the test more sensitive (90.9%, CI_95_ 70.8–98.9%), albeit non-significantly (*P* = 0.89), but as a consequence, specificity would drop significantly (88.4%, CI_95_ 87.3–89.4%, *P*<0.0001). The AUC for the hemastix test was 0.930 (CI_95_ 0.921–0.938, *P*<0.0001), with a Younden J index of 0.8024.

## Discussion

Results indicate that the proposed protocol can be executed in rural areas, successfully achieving the target sample size (17 896 of the targeted 18 180 school-going children) from a geographically representative area at reduced cost. Nevertheless, it is important to remember that the World Bank considers Namibia an Upper Middle-Income Country and therefore its rural areas may not be representative of other rural areas throughout Africa [47]. Therefore, we suggest that this protocol should be re-evaluated in a more representative country. With this in mind, due to the proximity to Namibia and existence of cross-border cooperation in public health, Angola is currently being mapped using the same protocol.

### Mapping resolutions

The concept of mapping resolution is introduced here in detriment of abstract methodologies of sample selection, such as “a number of schools per implementation unit” or “a number of schools per ecological zone”. For example, Namibia has eight ecological zones, and about 90% of the geographical area occupied by the six regions surveyed in this study falls within the Kalahari sands plateau ecological zone [48]. If we had followed WHO guidelines we would have ended up selecting only 5–10 schools in total, and would have likely lost important information regarding STH infections as they were not homogenously distributed across this ecological zone. The concept of mapping resolution can easily guide control managers from any country, whatever the dimension of population density with a simple adjustment of the ratio of sampled schools.

For Namibia, we suggest a higher mapping resolution of 1 in 4 schools for schistosomiasis as compared to 1 in 20 schools for STH infections. The sample size calculations were highly biased towards more power, consequently allowing for retrospective effectiveness analysis. A higher mapping resolution of 1 in 4 schools for schistosomiasis is of particular importance due to hot-spot identifications and heterogeneity of transmission [49], especially in a low transmission area such as this.

### Cost-effectiveness analysis

The total cost (in US Dollars) of mapping 17 896 children in 299 schools from northern Namibia using this protocol was $126,282.48, which included the cost of procurement (consumables, equipment and tests), communication expenses and per diems. This means that the average cost per child surveyed was $7.06, of which 49% was the cost of staff per diems. Furthermore, if both arms of the mapping are analyzed separately, the cost surveying each child using RDTs for schistosomiasis was $3.59 while the cost of mapping each child using standard microscopy techniques was $9.55. This difference was largely due to salaries and time spent in the field, as explained by previous work [24,50]. Our results underscore the need for a detailed, cost-comparative study between POC-RDTs and their homologous light microscopy techniques.

The proposed protocol is time-efficient, cost-effective, sensitive to budget limitations and the potential economic and logistical strains placed on the Ministry. This protocol reduces costs of mapping while at the same time increasing sample size without losing specificity for directed treatment of schools with the highest burden of disease.

### Water, sanitation and hygiene

All six regions covered in Phases 1 and 2 are mostly rural and far from the capital. Therefore, one would expect lower standards of water-availability, sanitation and hygiene. However, the numbers reported were encouraging with 75% of schools with latrines in good working condition and 88% of schools providing a safe water source to the students. On the other hand, and less encouraging, was the low coverage achieved by previous albendazole distribution campaigns which were below the 75% coverage threshold set by WHO. One aspect that could have hindered the performance of past campaigns is the administration of treatment at schools by nurses from constituency/regional level and not by the teachers. Namibia is advised to follow WHO guidelines for deworming at the school using teachers as drug administrators [36]. Data from this survey suggested that teachers would be very receptive to training and would like to be involved in such a campaign.

### Schistosomiasis and STH infections

Results show that schistosomiasis, although prevalent, does not reach alarming levels (overall prevalence of 9%) in Namibia. Nevertheless, it is important to note that of the 299 sampled schools, 64 (21%) had moderate transmission and 7 (2%) had heavy transmission. Therefore, these areas of Namibia, especially Caprivi and Kavango regions, need treatment and interventions such as drug administration. Within these high prevalence regions, there was a high degree of spatial heterogeneity that could only partially be explained by the environmental risk factors considered. Whilst at present, all schools in an area exceeding the 10% prevalence threshold will be targeted for preventive chemotherapy, more focalised strategies may be required to eliminate the disease. As such, a greater knowledge of the factors influencing the small scale spatial variability in prevalence is essential.

Schistosomiasis was equally present across our sampled population; however, there was a significantly higher probability of infection among older (OR = 1.03, P<0.001 for every additional year of age) boys (OR = 1.27, P<0.00001 when compared to girls). This was particularly evident in higher transmission areas. Given the overall low prevalence of disease, the treatment needs of pre-school children do not appear to be a priority. Nonetheless, if sufficient amounts of praziquantel were available, treatment consideration of these younger children could be explored in Kongola constituency, Caprivi region.

Overall, STH infections were largely absent with hookworms dominating as expected [1]. Only Kavango region recorded levels above 20%. However, Hookworm hot-spots were identified in a few constituencies in the Ohangwena region, such as Omundaungilo, Oshikango, Eenhana and Omulonga. Hookworm infections were more common in older (1.04, P = 0.042 for every additional year) girls (1.49, P<0.00001 when compared to boys). The latter point contradicts previous research and warrants further investigation [51].

### Morbidity markers

Intestinal morbidity was associated with Hookworm and *H*. *nana* infections in these regions of Namibia, but not with *S*. *mansoni* infection, unlike previous reports from other areas in Sub-Saharan Africa [31,32]. It is important to mention, however, that while Kavango region had the highest prevalence of hookworm infection, it had the second lowest prevalence of intestinal morbidity, meaning that while an association may exist between infection and blood in stool, there are definitely other factors at play. The levels of intestinal morbidity identified in Phase 2 of the mapping needs further investigation (average prevalence of 21.7% (CI_95_ 20.0–23.4%). One aspect which warrants consideration is the fact that Namibia was battling its worst drought for decades during the dry season of 2013 (while we were mapping). Drought-related chronic dehydration could have led to constipation and/or more compacted stools, which in turn could cause haemorrhoids, fissures and bleeding when stooling and hence positive stool samples containing blood [52].

### Treatment recommendations

**Fig 5:**
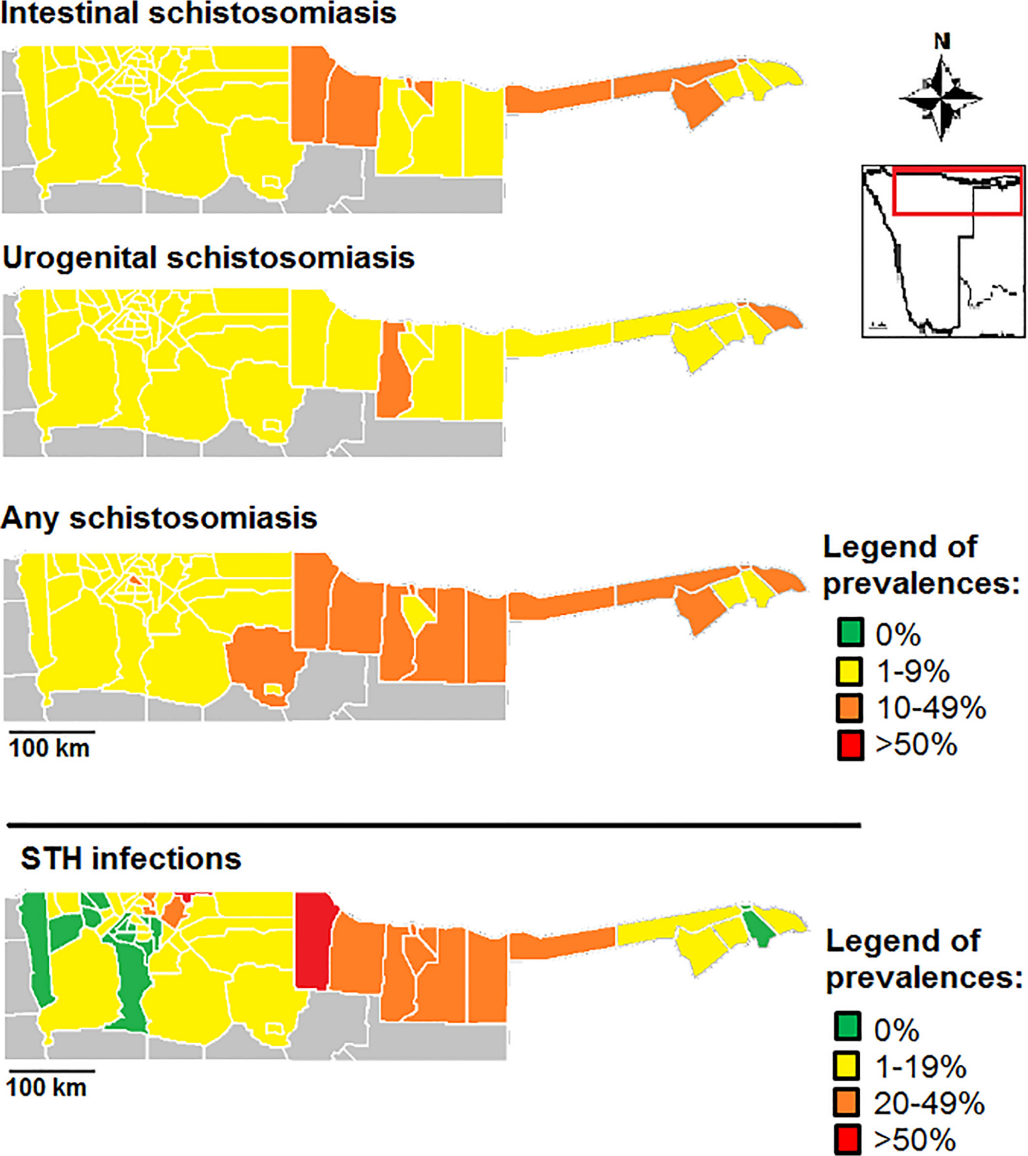
Prevalence of schistosomiasis (urogenital, intestinal and any type) and hookworm infections by constituency.

The data suggests that there is a significant need for treatment against schistosomiasis and STH infections in northern Namibia. Additionally, in some constituencies, school-based treatment campaigns should run in tandem with community-based campaigns related to treatment and hygiene and sanitation (especially provision of safe water at schools) (Table 4 and Fig 5).

**Table 4:** Treatment recommendations according to WHO guidelines.

	Recorded Prevalence	Praziquantel	Albendazole	WASH Improvements
Caprivi	Schistosomiasis 16%	Treat every two years	No mass treatment needed	none
	STH infections 4%			
Kavango	Schistosomiasis 18%	Treat every two years	Annual treatment *	Mpungu constituency
	STH infections 28%			
Ohangwena	Schistosomiasis 4%	Treatment at least once during primary school years	No mass treatment needed **	Omundaungilo constituency
	STH infections 16%	(e.g. every five years)		
Omusati	Schistosomiasis 5%	Treatment at least once during primary school years	No mass treatment needed	none
	STH infections 2%	(e.g. every five years)		
Oshana	Schistosomiasis 7%	Treatment at least once during primary school years	No mass treatment needed	none
	STH infections 5%	(e.g. every five years)		
Oshikoto	Schistosomiasis 3%	Treatment at least once during primary school years	No mass treatment needed	none
	STH infections 5%	(e.g. every five years)		

* Mpungu constituency could be treated twice yearly and include community-wide deworming at least once a year.

** Treatment could be considered for Omundaungilo (twice a year), Oshikango, Eenhana and Omulonga (once a year) at a constituency level.

WASH = Water, sanitation and hygiene

Although transmission varied between constituencies, a praziquantel and albendazole distribution campaign should use the regions as implementation units for the first five years, especially given the focalized risk for schistosomiasis. After five years, the implementation unit may then be downscaled to the constituency-level as some areas will be free of infection while others will need continued deworming.

Due to the overall good school-enrolment numbers and obvious political will in Namibia, this survey suggests that a successful school-based campaign will have tremendous impact on burden of schistosomiasis and STH infections. The fact that Namibia lies on the southern fringe of the schistosomiasis-endemic regions of Africa, experiences seasonal transmission, has a low morbidity prevalence, and has low to moderate transmission puts northern Namibian regions in a great position to be one of the first areas in Africa to successfully eliminate schistosomiasis.

### Conclusion

The overall low risk and the highly focal transmission of schistosomiasis make northern Namibia a "low hanging fruit" for transmission elimination with mass drug administration alone. Importantly, not only did levels of schistosomiasis not reach alarming levels (>50%) at a constituency level, heavy schistosomiasis infections were uncommon with only a few constituencies being highlighted as problematic. Nevertheless, these deceptively low levels of schistosomiasis cover up hotspots which reached close to 80%. These focal areas are where burden of disease will be highest and harder to eliminate and may require a multidisciplinary approach. Our study served as a first proving test for a new type of mapping protocols and identified the enormous potential of POC-RDTs to be regularly employed as a more cost-effective diagnostic technique in large mapping surveys.

## Supporting Information

S1 Checklist STARD checklist. (DOC)

S1 Flowchart STARD flowchart for CCA. (TIF)

S2 Flowchart STARD flowchart for Hemastix. (TIF)

S1 Table Sample size estimations for Namibia for Phases 1 and 2 separately and in total. (DOCX)

S2 Table Numbers of children surveyed by age. (DOCX)

S1 Fig Questionnaire results by region and in total. (TIF)

S2 Fig ROC curve for Model 1, plus the sensitivity of the model with respect to a range of prevalence thresholds between 0 and 100%. (TIF)

S3 Fig ROC curves of the urine CCA test (above) and the Hemastix (below) using light microscopy (Kato-Katz and urine filtration techniques, respectively) as the ‘field-standard’. The receiver operating characteristic (ROC) curves, the area under the curve (AUC) and the standard error (Strd. Err) of the CCA and the Hemastix tests are presented.
(TIF)
