# Supplementary figures and images for: Mapping of Schistosomiasis and Soil-Transmitted Helminths in Namibia: The First Large-Scale Protocol to Formally Include Rapid Diagnostic Tests
Source: PLoS Negl Trop Dis. 2015 Jul 21;9(7):e0003831. doi: 10.1371/journal.pntd.0003831 (PMC4509651; doi:10.1371/journal.pntd.0003831)

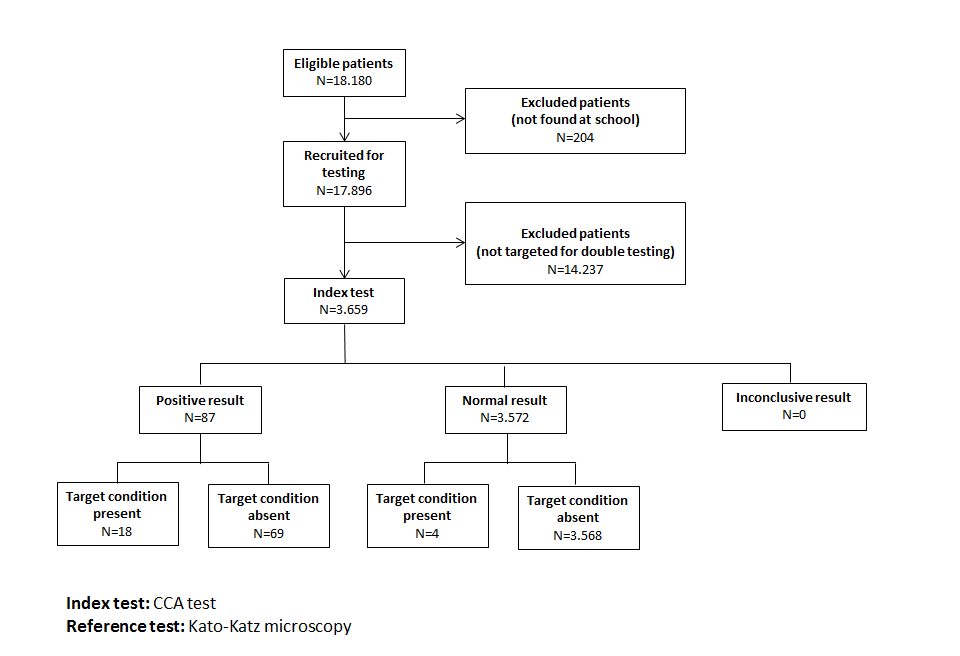

Supplement: S1 Flowchart — (TIF) [file pntd.0003831.s002.tif]

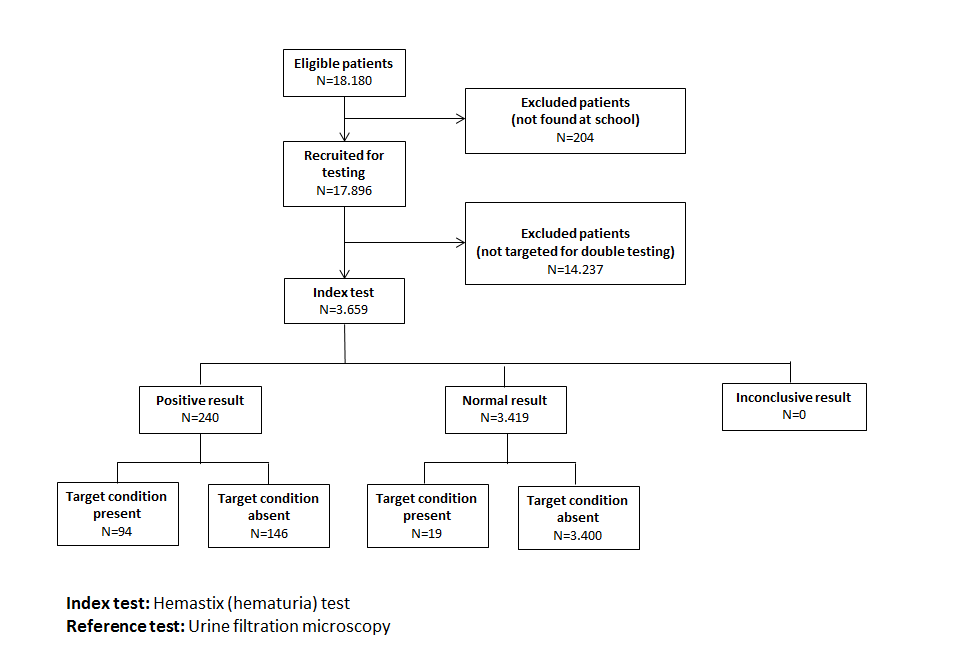

Supplement: S2 Flowchart — (TIF) [file pntd.0003831.s003.tif]

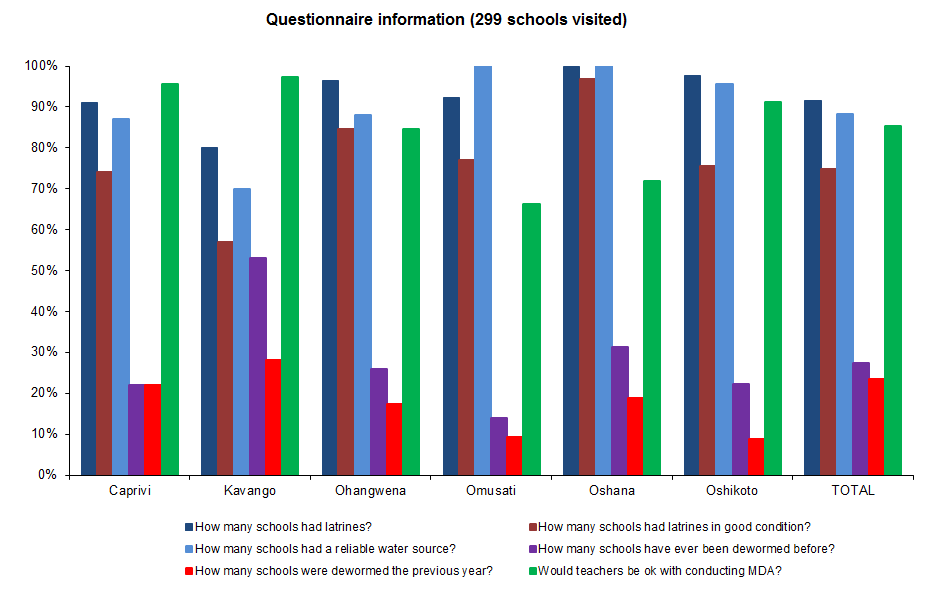

Supplement: S1 Fig — (TIF) [file pntd.0003831.s006.tif]

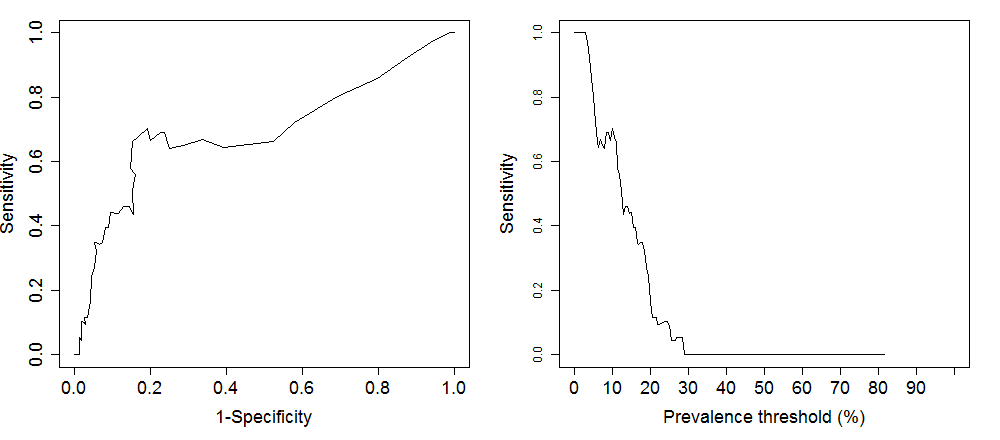

Supplement: S2 Fig — (TIF) [file pntd.0003831.s007.tif]

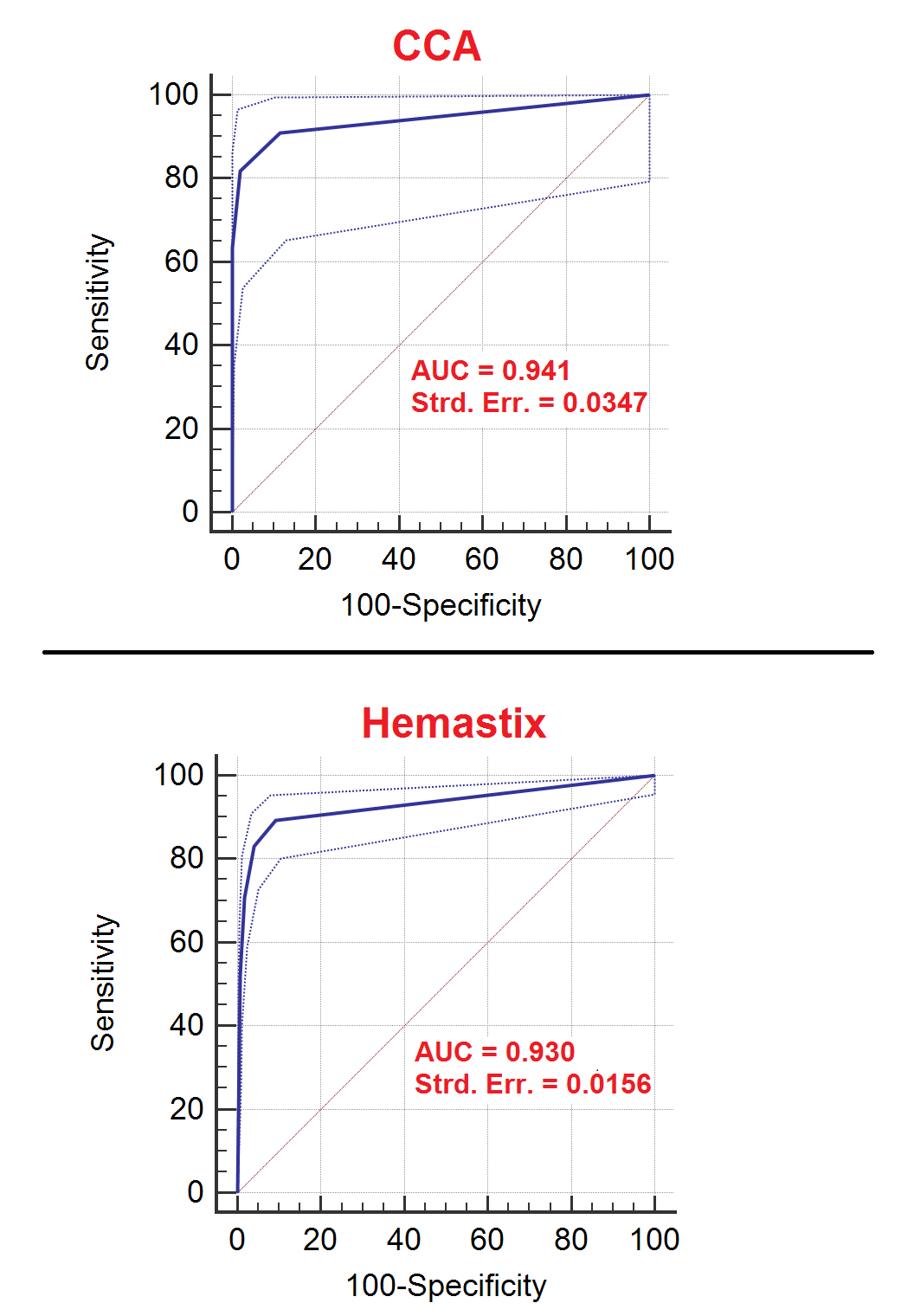

Supplement: S3 Fig — The receiver operating characteristic (ROC) curves, the area under the curve (AUC) and the standard error (Strd. Err) of the CCA and the Hemastix tests are presented. (TIF) [file pntd.0003831.s008.tif]
