# Supplementary material for: Mapping of Schistosomiasis and Soil-Transmitted Helminths in Namibia: The First Large-Scale Protocol to Formally Include Rapid Diagnostic Tests
Source: PLoS Negl Trop Dis. 2015 Jul 21;9(7):e0003831. doi: 10.1371/journal.pntd.0003831 (PMC4509651; doi:10.1371/journal.pntd.0003831)
Supplement: S1 Table — (DOCX) [file pntd.0003831.s004.docx]

| **Item** | **Age-classes** | **Phase 1** | | **Phase 2** | | **Total** | |
| --- | --- | --- | --- | --- | --- | --- | --- |
|  |  | **RDT only** | **Microscopy and RDT** | **RDT only** | **Microscopy and RDT** | **RDT only** | **Microscopy and RDT** |
| Total no. schools available* | | 397 | | 829 | | 1226 | |
| No. schools to be surveyed | | 79 | 20 | 163 | 41 | 242 | 61 |
| Total no. of schools to be surveyed | | 99 | | 204 | | 303 | |
| No. children to be surveyed | | 4740 | 1200 | 9780 | 2460 | 14520 | 3660 |
|  | First grade | 2370 | 600 | 4890 | 1230 | 7260 | 1830 |
|  | Later grades | 2370 | 600 | 4890 | 1230 | 7260 | 1830 |
| Total no. of children to be surveyed | | 5940 | | 12240 | | 18180 | |

* 2012 MoE data for Phase 1 and 2013 MoE data for Phase 2
