# Supplementary material for: Mapping of Schistosomiasis and Soil-Transmitted Helminths in Namibia: The First Large-Scale Protocol to Formally Include Rapid Diagnostic Tests
Source: PLoS Negl Trop Dis. 2015 Jul 21;9(7):e0003831. doi: 10.1371/journal.pntd.0003831 (PMC4509651; doi:10.1371/journal.pntd.0003831)
Supplement: S2 Table — (DOCX) [file pntd.0003831.s005.docx]

| **Ages (years)** | **Rapid diagnostics** | **Microscopy** |
| --- | --- | --- |
| 3 | 5 | 0 |
| 4 | 20 | 3 |
| 5 | 174 | 46 |
| 6 | 1 578 | 247 |
| 7 | 3 714 | 736 |
| 8 | 2 049 | 419 |
| 9 | 1 207 | 227 |
| 10 | 2 381 | 600 |
| 11 | 1 979 | 459 |
| 12 | 1 854 | 344 |
| 13 | 1 679 | 337 |
| 14 | 1 026 | 165 |
| 15 | 168 | 52 |
| 16 | 37 | 19 |
| 17 | 15 | 3 |
| 18 | 8 | 2 |
| 19 | 2 | 0 |
| TOTAL | 17 896 | 3 659 |
